# Obesity-Related Genomic Loci Are Associated with Type 2 Diabetes in a Han Chinese Population

**DOI:** 10.1371/journal.pone.0104486

**Published:** 2014-08-05

**Authors:** Xiaomu Kong, Xuelian Zhang, Qi Zhao, Jiang He, Li Chen, Zhigang Zhao, Qiang Li, Jiapu Ge, Gang Chen, Xiaohui Guo, Juming Lu, Jianping Weng, Weiping Jia, Linong Ji, Jianzhong Xiao, Zhongyan Shan, Jie Liu, Haoming Tian, Qiuhe Ji, Dalong Zhu, Zhiguang Zhou, Guangliang Shan, Wenying Yang

**Affiliations:** 1 Department of Endocrinology, Key Laboratory of Diabetes Prevention and Control of China-Japan Friendship Hospital, Beijing, China; 2 Department of Epidemiology, Tulane School of Public Health and Tropical Medicine, New Orleans, Louisiana, United States of America; 3 Department of Endocrinology, Qilu Hospital of Shandong University, Jinan, Shandong, China; 4 Department of Endocrinology, Henan Province People's Hospital, Zhengzhou, Henan, China; 5 Department of Endocrinology, Second Affiliated Hospital of Harbin Medical University, Harbin, Heilongjiang, China; 6 Department of Endocrinology, Xinjiang Uygur Autonomous Region's Hospital, Urmqi, Xinjiang, China; 7 Department of Endocrinology, Fujian Provincial Hospital, Fuzhou, Fujian, China; 8 Department of Endocrinology, Peking University First Hospital, Beijing, China; 9 Department of Endocrinology, Chinese People's Liberation Army General Hospital, Beijing, China; 10 Department of Endocrinology, Sun Yat-sen University Third Hospital, Guangzhou, Guangdong, China; 11 Department of Endocrinology, Shanghai Jiaotong University Affiliated Sixth People's Hospital, Shanghai, China; 12 Department of Endocrinology, Peking University People's Hospital, Beijing, China; 13 Department of Endocrinology, First Affiliated Hospital, Chinese Medical University, Shenyang, Liaoning, China; 14 Department of Endocrinology, Shanxi Province People's Hospital, Taiyuan, Shanxi, China; 15 Department of Endocrinology, West China Hospital, Sichuan University, Chengdu, Sichuan, China; 16 Department of Endocrinology, Xijing Hospital, Fourth Military Medical University, Xi'an, Shaanxi, China; 17 Department of Endocrinology, Affiliated Drum Tower Hospital of Nanjing University Medical School, Nanjing, Jiangsu, China; 18 Department of Endocrinology, Xiangya Second Hospital, Changsha, Hunan, China; 19 Department of Epidemiology, Peking Union Medical College, Beijing, China; Wake Forest School of Medicine, United States of America

## Abstract

**Background and Aims:**

Obesity is a well-known risk factor for type 2 diabetes. Genome-wide association studies have identified a number of genetic loci associated with obesity. The aim of this study is to examine the contribution of obesity-related genomic loci to type 2 diabetes in a Chinese population.

**Methods:**

We successfully genotyped 18 obesity-related single nucleotide polymorphisms among 5338 type 2 diabetic patients and 4663 controls. Both individual and joint effects of these single nucleotide polymorphisms on type 2 diabetes and quantitative glycemic traits (assessing β-cell function and insulin resistance) were analyzed using logistic and linear regression models, respectively.

**Results:**

Two single nucleotide polymorphisms near *MC4R* and *GNPDA2* genes were significantly associated with type 2 diabetes before adjusting for body mass index and waist circumference (OR (95% CI) = 1.14 (1.06, 1.22) for the A allele of rs12970134, *P* = 4.75×10^−4^; OR (95% CI) = 1.10 (1.03, 1.17) for the G allele of rs10938397, *P* = 4.54×10^−3^). When body mass index and waist circumference were further adjusted, the association of *MC4R* with type 2 diabetes remained significant (*P* = 1.81×10^−2^) and that of *GNPDA2* was attenuated (*P* = 1.26×10^−1^), suggesting the effect of the locus including *GNPDA2* on type 2 diabetes may be mediated through obesity. Single nucleotide polymorphism rs2260000 within *BAT2* was significantly associated with type 2 diabetes after adjusting for body mass index and waist circumference (*P* = 1.04×10^−2^). In addition, four single nucleotide polymorphisms (near or within *SEC16B*, *BDNF*, *MAF* and *PRL* genes) showed significant associations with quantitative glycemic traits in controls even after adjusting for body mass index and waist circumference (all *P* values<0.05).

**Conclusions:**

This study indicates that obesity-related genomic loci were associated with type 2 diabetes and glycemic traits in the Han Chinese population.

## Introduction

The prevalence of diabetes has increased dramatically in China during the past few decades. According to the recent Chinese National Diabetes and Metabolic Disorders Study (DMS) performed in 2007–2008, approximately 92.4 million adults in China had diabetes [1]. Type 2 diabetes, the most common type of diabetes, is determined by both genetic and environmental factors [2], [3]. Understanding the genetic mechanism underlying the pathogenesis of type 2 diabetes will be critical for the prevention and treatment of type 2 diabetes. Although remarkable progress has been made in elucidating type 2 diabetes genetic mechanisms by recent genome-wide association studies (GWAS), a large proportion of heritability of this disease is still unclear [2]–[4].

Obesity is a well-established risk factor for type 2 diabetes [4]–[8]. Recently, a number of genomic loci have been identified as being associated with obesity or obesity-related quantitative traits, such as body weight, body mass index (BMI), and waist circumference (WC) by GWAS [9]. Some of these variants also contribute to the risk of type 2 diabetes [10]–[28]. For example, *FTO* is among the genes reported to be associated with both disorders [23], [24], [26]. However, inconsistent study findings regarding the associations between some obesity-related genomic loci and type 2 diabetes risk have been reported in Han Chinese [11]–[14], [29], [30], who constitute more than 90% of the population of China. In addition, the roles of these genetic variants in insulin resistance and β-cell function are still unclear.

The aim of the present study is to examine individual and joint effects of obesity-related genomic loci on the risk of type 2 diabetes and quantitative assessments of insulin resistance and β-cell function in a large Han Chinese population comprised of 5338 patients with type 2 diabetes and 4663 controls.

## Materials and Methods

### Ethics statement

The study protocol was approved by the Ethics Committee of China-Japan Friendship Hospital in Beijing and was in accordance with the Helsinki Declaration II. Written informed consents were obtained from all participants before data collection.

### Study subjects

All the study subjects were from the DMS [1]. A total of 5338 type 2 diabetes cases and 4663 controls were included in the study. Type 2 diabetes cases were identified using the 1999 WHO criteria, including fasting plasma glucose (FPG) ≥7.0 mmol/l, 2-h oral glucose tolerance test (OGTT) plasma glucose ≥11.1 mmol/l, or a self-reported history of type 2 diabetes. A random sample of 4663 participants without type 2 diabetes or pre-diabetes (FPG<6.1 mmol/l and 2-h OGTT plasma glucose <7.8 mmol/l) from the DMS were included as controls.

### Measurements

Body weight, height, and WC were measured using standard methods. Each participant completed a standard 75 g OGTT after overnight fasting. Blood samples were drawn at 0 minutes, 30 minutes, and 2 hours after OGTT to measure plasma glucose and serum insulin concentrations. Serum insulin was measured by double-antibody radioimmunoassay. HOMA-B and insulinogenic index were calculated to estimate β-cell function, and HOMA-IR and Matsuda index (ISIm) were used to assess insulin resistance. The formulas are described below:
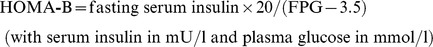


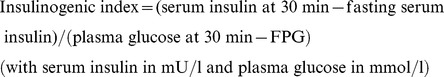


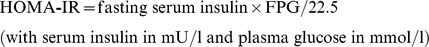


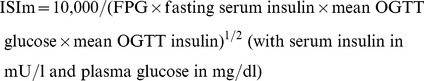



### Genotyping

Genomic DNA samples were isolated from the peripheral blood using a DNA extraction kit. We selected 25 single nucleotide polymorphisms (SNPs) from 24 genetic loci which were identified as being associated with BMI, body weight, WC, or obesity status by previous GWAS [9]. Genotyping was performed using the Illumina GoldenGate Indexing assay (Illumina Inc., San Diego, USA) according to the manufacturer's instructions. We excluded SNPs with genotyping call rates <85% (rs7498665 from *SH2B1* and rs11084753 near *KCTD15*) or minor allele frequency (MAF) <1% (rs10508503 near *PTER*, rs6232 in *PCSK1*, rs6602024 in *PFKP*, rs6013029 in *CTNNBL1*, and rs10146997 in *NRXN3*). The average genotyping call rate of the remaining 18 SNPs was 96.54%, with a concordance rate of 100% based on 229 genotyping duplications. Information of the 18 SNPs is listed in Table S1.

### Statistical analyses

The Hardy-Weinberg equilibrium test was performed for each SNP using a χ^2^ test in the control sample. All non-Gaussian distributed quantitative traits in the control group were natural logarithmically transformed to normalize distributions. An additive genetic model was assumed. Logistic and linear regression models were used to test the associations of SNPs with type 2 diabetes and quantitative glycemic traits, respectively. Three multivariable models were tested: in model 1, age and sex were adjusted as co-variables; in model 2, age, sex, and BMI were adjusted; and in model 3, age, sex, BMI, and WC were adjusted [22]. In addition to the individual SNP test, we also analyzed the joint effects of these SNPs on the risk of type 2 diabetes and quantitative glycemic traits. A genetic risk score of obesity-associated SNPs was constructed using the sum of alleles which were reported as risk alleles for obesity in each individual without missing data [24]. The risk for type 2 diabetes was compared among quartiles of the genetic risk score. The effects of the genetic risk score on quantitative traits were also examined.

Meta-analysis was conducted to combine our current findings with previous studies of two significant SNPs near *MC4R* and *GNPDA2* among Han Chinese populations. Cochran's χ^2^-based Q-statistic test was performed to assess heterogeneity across all of the included studies. Fixed-effects models were used to calculate the pooled ORs. The inverse variance was used to weight each study. The significant *P* values for ORs were determined using a Z-test.

Statistical analyses were performed using SAS (version 9.3; SAS Institute, Cary, NC) and PLINK software (v1.05). We also assessed the study power using QUANTO software [11] (available at http://hydra.usc.edu/gxe/). For the case-control sample, we had a statistical power of >80% to detect an OR of 1.10 for risk allele frequencies ranging between 25% and 75%.

## Results

The clinical characteristics of type 2 diabetes cases and controls of the DMS are shown in Table 1. Overall, the case group was older and had greater weight, BMI, and WC than the control group (all *P* values<0.0001). In addition, cases showed higher glucose levels during fasting and the OGTT (all *P* values<0.0001) and worse assessments of β-cell function and insulin resistance (all *P* values<0.0001 except for *P* = 0.0032 for Insulinogenic index). All of the 18 analyzed SNPs followed Hardy-Weinberg equilibrium in control subjects, except for rs10913469 near *SEC16B* (*P* = 2.03×10^−3^) (Table S1). The MAFs of the genotyped SNPs in the present study were close to those reported for Han Chinese in Beijing in the HapMap project (Table S1).

**Table 1:** Clinical characteristics of study population.

	Type 2 diabetes	Control	*P*
N	5338	4663	
Male, %	43.3	32.2	<0.0001
Age, year	56.0 (47.0, 64.0)	49.0 (44.0, 56.0)	<0.0001
Body weight, kg	66.1 (59.0, 75.0)	59.1 (54.0, 65.0)	<0.0001
BMI, kg/m^2^	25.6 (23.4, 28.2)	23.1 (21.3, 24.8)	<0.0001
Waist circumference, cm	88.0 (81.0, 95.0)	79.0 (73.0, 85.0)	<0.0001
Fasting plasma glucose, mmol/l	7.3 (6.2, 9.0)	5.0 (4.7, 5.4)	<0.0001
30-min OGTT glucose, mmol/l	11.9 (9.8, 14.3)	8.1 (7.0, 9.2)	<0.0001
2-h OGTT glucose, mmol/l	13.4 (11.2, 17.0)	5.8 (4.9, 6.6)	<0.0001
Fasting serum insulin, mU/l	8.7 (6.1, 12.6)	6.3 (4.9, 8.5)	<0.0001
30-min OGTT insulin, mU/l	20.1 (11.5, 36.3)	32.9 (21.0, 52.5)	<0.0001
2-h OGTT insulin, mU/l	32.2 (18.6, 60.4)	22.2 (13.7, 35.0)	<0.0001
HOMA-B, %	47.0 (28.0, 77.0)	85.5 (61.0,125.6)	<0.0001
Insulinogenic index	2.9 (1.3, 6.3)	9.4 (5.0, 17.3)	0.0032
HOMA-IR	3.0 (1.9, 4.6)	1.4 (1.1, 1.9)	<0.0001
ISIm	4.3 (2.9, 6.3)	8.4 (6.2, 11.3)	<0.0001

Abbreviations: BMI, body mass index; OGTT, oral glucose tolerance test; ISIm, Matsuda index.

Data are shown as median (interquartile range) or %.

### Associations between obesity-related SNPs and type 2 diabetes

Table 2 shows the associations between individual SNPs and type 2 diabetes in the study population. After adjustment for age and sex, two SNPs near the *MC4R* (rs12970134) and *GNPDA2* (rs10938397) genes were found to be significantly associated with type 2 diabetes (*P* = 4.75×10^−4^ and 4.54×10^−3^, respectively). The risk alleles of these two SNPs for type 2 diabetes were consistent with the risk alleles for obesity. The association between rs12970134 and type 2 diabetes remained significant even after additional adjustment for BMI (*P* = 1.53×10^−2^) or both BMI and WC (*P* = 1.81×10^−2^). The association of rs10938397 with type 2 diabetes was abolished after adjusting for BMI (*P*>0.05). In addition, rs2260000 within the *BAT2* gene was significantly associated with type 2 diabetes only after adjusting for BMI (*P* = 4.96×10^−3^) or both BMI and WC (*P* = 1.04×10^−2^). The T allele of this SNP was significantly associated with greater body weight (*P* = 1.04×10^−4^) and BMI (*P* = 1.52×10^−3^) in the studied DMS sample. However, the risk allele for diabetes of this SNP (allele C) was not consistent with that for obesity (allele T).

**Table 2:** Associations between obesity-related SNPs and type 2 diabetes in the study population.

SNP	Gene	Chr	Major/minor allele^a^	Model 1		Model 2		Model 3	
				OR (95%CI)^b^	*P*	OR (95%CI)^b^	*P*	OR (95%CI)^b^	*P*
rs2568958	*NEGR1*	1	A/G	0.96 (0.87,1.07)	4.70E-01	0.98 (0.88,1.10)	7.73E-01	0.98 (0.87,1.11)	7.85E-01
rs10913469	*SEC16B*	1	T/C	1.03 (0.96,1.11)	3.60E-01	0.99 (0.91,1.07)	7.82E-01	1.00 (0.92,1.08)	9.18E-01
rs2605100	*SLC30A10*	1	G/A	0.95 (0.89,1.02)	1.36E-01	0.93 (0.87,1.01)	8.21E-02	0.93 (0.86,1.01)	6.79E-02
rs7561317	*TMEM18*	2	G/A	0.92 (0.84,1.02)	1.13E-01	0.92 (0.83,1.03)	1.56E-01	0.92 (0.82,1.03)	1.38E-01
rs7647305	*ETV5/DGKG*	3	C/T	0.90 (0.80,1.01)	8.19E-02	0.90 (0.79,1.03)	1.14E-01	0.91 (0.80,1.04)	1.84E-01
rs10938397	*GNPDA2*	4	A/G	1.10 (1.03,1.17)	**4.54E-03**	1.06 (0.99,1.14)	9.02E-02	1.06 (0.98,1.14)	1.26E-01
rs2260000	*BAT2*	6	C/T	0.95 (0.90,1.01)	1.06E-01	0.91 (0.85,0.97)	**4.96E-03**	0.92 (0.86,0.98)	**1.04E-02**
rs4712652	*PRL*	6	A/G	0.99 (0.91,1.07)	7.39E-01	0.99 (0.91,1.09)	8.54E-01	0.99 (0.90,1.08)	7.61E-01
rs987237	*TFAP2B*	6	A/G	0.96 (0.89,1.04)	3.23E-01	0.96 (0.88,1.04)	3.14E-01	0.95 (0.87,1.04)	2.87E-01
rs545854	*MSRA*	8	G/C	1.00 (0.95,1.07)	9.02E-01	1.02 (0.95,1.08)	6.40E-01	1.02 (0.95,1.09)	6.01E-01
rs4923461	*BDNFOS*	11	A/G	0.98 (0.93,1.04)	5.20E-01	0.99 (0.93,1.05)	6.94E-01	0.99 (0.92,1.05)	6.79E-01
rs925946	*BDNF*	11	G/T	0.98 (0.86,1.12)	7.63E-01	0.95 (0.82,1.10)	4.92E-01	0.96 (0.83,1.11)	5.56E-01
rs10838738	*MTCH2*	11	A/G	1.04 (0.98,1.11)	1.71E-01	1.03 (0.96,1.10)	4.28E-01	1.03 (0.96,1.10)	4.76E-01
rs7138803	*FAIM2*	12	G/A	1.03 (0.97,1.10)	3.84E-01	1.00 (0.94,1.08)	9.04E-01	1.01 (0.94,1.08)	8.24E-01
rs1424233	*MAF*	16	A/G	0.98 (0.92,1.05)	5.79E-01	0.97 (0.91,1.04)	4.63E-01	0.98 (0.92,1.05)	6.32E-01
rs12970134	*MC4R*	18	G/A	1.14(1.06,1.22)	**4.75E-04**	1.10 (1.02,1.20)	**1.53E-02**	1.10 (1.02,1.20)	**1.81E-02**
rs1805081	*NPC1*	18	A/G	0.97 (0.91,1.04)	4.28E-01	0.97 (0.90,1.05)	4.20E-01	0.98 (0.91,1.06)	5.98E-01
rs29941	*KCTD15*	19	T/C	1.01 (0.94,1.08)	7.87E-01	1.00 (0.93,1.09)	9.20E-01	1.00 (0.92,1.08)	9.22E-01

Abbreviations: SNP, single nucleotide polymorphism; Chr, chromosome; OR, odds ratio; CI, confidence interval; BMI, body mass index; WC, waist circumference.

a. Risk allele for obesity is underlined.

b. OR and 95% CI are reported for the minor allele of each SNP using logistic regression under an additive assumption using the following models: model 1, adjusted for age and sex; model 2, adjusted for age, sex and BMI; and model 3, adjusted for age, sex, BMI and WC.

*P* values<0.05 are shown in bold.

Joint effect analyses showed that the genetic risk score of obesity-associated SNPs was significantly associated with the risk of type 2 diabetes (Table 3). Individuals with more obesity risk alleles had greater risk for type 2 diabetes. Compared to the lowest quartile of the genetic risk score, the ORs (95% CI) were 1.24 (1.08, 143), 1.19 (1.03, 1.37), and 1.29 (1.10, 1.51) for the other three quartiles (*P* for trend = 4.80×10^−3^). The association was eliminated after additional adjustment for BMI or BMI and WC (both *P* values>0.05).

**Table 3:** Joint effects of obesity-related risk alleles on the risk for type 2 diabetes in study population.

Quartile	Model 1		Model 2		Model3	
	OR (95%CI)	*P*	OR (95%CI)	*P*	OR (95%CI)	*P*
Q1	1		1		1	
Q2	1.24 (1.08,1.43)	**2.60E-03**	1.28 (1.10,1.50)	**2.00E-03**	1.28 (1.09,1.50)	**2.10E-03**
Q3	1.19 (1.03,1.37)	**1.87E-02**	1.19 (1.01,1.39)	**3.37E-02**	1.17 (0.99,1.37)	5.90E-02
Q4	1.29 (1.10,1.51)	**1.40E-03**	1.18 (1.00,1.41)	5.72E-02	1.18 (0.99,1.40)	6.85E-02
*P*_trend_	**4.80E-03**	1.21E-01	1.66E-01			

Abbreviations: OR, odds ratio; CI, confidence interval; BMI, body mass index; WC, waist circumference.

OR and 95% CI are reported for genotype risk score quartiles using logistic regression under the following models: model 1, adjusted for age and sex; model 2, adjusted for age, sex and BMI; and model 3, adjusted for age, sex, BMI and WC.

*P* values<0.05 are shown in bold.

### Associations between obesity-related SNPs and glycemic traits

We identified 4 SNPs as being significantly associated with quantitative glycemic traits (Table 4). Except for SNP rs925946 close to the *BDNF* gene, the obesity risk alleles of these SNPs were not consistent with the risk alleles associated with dysregulated glycemic traits. For example, the obesity-related risk allele (A) of SNP rs1424223 near the *MAF* gene was associated with a lower 30-min glucose level during the OGTT. In addition, the obesity-related risk allele (A) of SNP rs4712652 in the *PRL* gene was associated with a lower 2-h glucose level during the OGTT and a higher level of β cell function. All the associations remained significant after adjusting for BMI and WC. We did not observe any significant associations of the three type 2 diabetes-associated SNPs (rs12970134 near *MC4R*, rs10938397 near *GNPDA2*, and rs2260000 within *BAT2*) with these quantitative glycemic traits (all *P* values>0.05). We also did not observe any significant effect of the genetic risk score of obesity-associated SNPs on these quantitative glycemic traits (Table S2).

**Table 4:** Associations between obesity-related SNPs and glycemic traits in control subjects.

Traits	Gene	SNP	Major/minor allele^a^	Model 1		Model 2		Model 3	
				*β*(SE)^b^	*P*	*β*(SE)^b^	*P*	*β*(SE)^b^	*P*
30-min OGTT glucose	*MAF*	rs1424233	A/G	0.0042(0.0021)	4.71E-02	0.0042(0.0021)	4.92E-02	0.0042(0.0021)	4.66E-02
2-h OGTT glucose	*PRL*	rs4712652	A/G	0.0060(0.0026)	1.97E-02	0.0063(0.0026)	1.38E-02	0.0062(0.0026)	1.47E-02
30-min OGTT insulin	*BDNF*	rs925946	G/T	−0.0351(0.0154)	2.25E-02	−0.0346(0.0152)	2.26E-02	−0.0335(0.0152)	2.72E-02
2-h OGTT insulin	*PRL*	rs4712652	A/G	0.0203(0.0095)	3.21E-02	0.0228(0.0094)	1.51E-02	0.0229(0.0094)	1.44E-02
HOMA-B	*PRL*	rs4712652	A/G	−0.0199(0.0083)	1.64E-02	−0.0197(0.0083)	1.76E-02	−0.0196(0.0083)	1.78E-02
Insulinogenic index	*SEC16B*	rs10913469	T/C	0.0325(0.0129)	1.15E-02	0.0294(0.0128)	2.15E-02	0.0296(0.0128)	2.07E-02

Abbreviations: SNP, single nucleotide polymorphism; OGTT, oral glucose tolerance test; BMI, body mass index; WC, waist circumference.

a. Risk alleles for obesity are underlined.

b. All non-Gaussian distributed quantitative traits in the control group were natural logarithmically transformed to normalize distributions. *β* values are reported for the minor allele using linear regression under an additive assumption using the following models: model 1, adjusted for age and sex; model 2, adjusted for age, sex and BMI; and model 3, adjusted for age, sex, BMI and WC.

Associations with *P* value<0.05 are shown in the table.

### Meta-analysis of the associations of SNPs near MC4R and GNPDA2 with type 2 diabetes in Chinese populations

We identified five additional studies [12]–[14], [29], [30] which examined the associations of rs12970134 or its proxy SNP rs17782313 near *MC4R* with type 2 diabetes and two additional studies [11], [13] examining the associations of rs10938397 near *GNPDA2* with type 2 diabetes, all in populations of Han Chinese (Table 5). There were no additional studies of rs2260000 within *BAT2* in Han Chinese population. We combined the effects of rs12970134 and rs10938397 on type 2 diabetes reported in these studies with those of the present study using meta-analysis. No heterogeneity was observed among these studies (*P* for heterogeneity >0.05). The meta-analysis showed that both of the two SNPs were significantly associated with type 2 diabetes in Han Chinese before adjusting for BMI. However, the association of rs10938397 with type 2 diabetes was eliminated after adjusting for BMI (Table 5). These results were consistent with our study.

**Table 5:** Meta-analysis of the association between SNPs near *MC4R*, *GNPDA2* and risk for type 2 diabetes risk in Chinese populations.

Gene	SNP	Study (year)^ref^	Sample size (T2D/Control)	Meta-analysis^a^				Meta-analysis^b^			
				OR (95%CI)	*P*	I^2^	*P_heter_*	OR (95%CI)	*P*	I^2^	*P_heter_*
*MC4R*	rs12970134	Present study (2014)	5448/4663	1.14 (1.06,1.22)				1.10 (1.02,1.20)			
	rs12970134	Ng et al (2010) [13]	6013/1692	1.06 (0.96,1.17)				1.04 (0.93,1.17)			
	rs17782313	Tao et al (2012) [30]	1360/3098	1.09 (0.97,1.22)				0.93 (0.83,1.05)			
	rs17782313	Huang et al (2011) [12]	591/1200	0.95 (0.82,1.11)				-			
	rs17782313	Shi et al (2010) [29]	885/2076	1.20 (1.05,1.37)				1.04 (0.90,1.21)			
	rs17782313	Wen et al (2010) [14]	1165/1136	1.20 (1.04,1.39)				1.18 (1.01,1.37)			
		**Meta-analysis**	15 462/13 865^c^	1.11 (1.06,1.16)	**1.98E-06**	37.30%	1.58E-01	1.06 (1.00,1.11)	**3.61E-02**	47.40%	1.07E-01
*GNPDA2*	rs10938397	Present study (2014)	5448/4663	1.10 (1.03,1.17)				1.06 (0.99,1.14)			
	rs10938397	Han et al (2013) [11]	1117/1113	1.02 (0.89,1.17)				1.02 (0.90,1.17)			
	rs10938397	Ng et al (2010) [13]	6013/1692	1.10 (1.01,1.20)				1.04 (0.94,1.14)			
		**Meta-analysis**	12 578/7468	1.09 (1.04,1.14)	**4.44E-04**	0.00%	5.98E-01	1.05 (0.99,1.10)	8.04E-02	0.00%	8.66E-01

Abbreviations: SNP, single nucleotide polymorphism; T2D, type 2 diabetes; OR, odds ratio; CI, confidence interval; BMI, body mass index.

The study combined data of both rs12970134 and its proxy rs17782313 which are known in high linkage disequilibrium (r^2^ = 0.850 in a combined population of Chinese Han and Japanese, as reported in the 1000 Genome Project).

a. Without adjustment for BMI;

b. with adjustment for BMI;

c. sample size for *MC4R* result with adjustment for BMI is 14 871/12 665, since data from Huang et al [12] was not provided.

*P* values<0.05 are shown in bold.

## Discussion

In the present study, we identified the association of obesity-related SNPs, rs12970134 near *MC4R*, rs10938397 near *GNPDA2*, and rs2260000 within *BAT2*, with the susceptibility of type 2 diabetes in a large Han Chinese population. We also found that genetic variants near or within *SEC16B*, *BDNF*, *MAF* and *PRL* genes were significantly associated with quantitative glycemic traits in Chinese.

Obesity and type 2 diabetes are highly prevalent worldwide [1], [4], [7]. Obesity-associated insulin resistance is a major risk factor leading to type 2 diabetes [4], [6], [8]. Evidence has shown that genetic loci related to obesity could contribute to the risk for type 2 diabetes [10], [11], [13]–[16], [18], [20]–[26]. For example, allele A of SNP rs9939609 in the *FTO* gene was reported to be associated with both increased BMI in various populations and elevated risk for type 2 diabetes [31]–[33]. During recent decades, genetic studies have identified multiple susceptible genetic loci related to obesity [2], [9]. Although many studies have attempted to investigate the relationship between some obesity-related genetic loci and type 2 diabetes in different ethnicities, their associations are still far from fully understood [11]–[27], [34]. Notably, previous studies conducted in Chinese populations have shown inconsistent results [11]–[14], [29], [30]. Thus, it is worthwhile to examine the associations between obesity-related SNPs and type 2 diabetes in a large sample of a Han Chinese population.

The gene product of *MC4R*, melanocortin 4 receptor, is a key link between the central nervous system and energy balance via the hypothalamic leptin-melanocortin pathway [35]. Mutation in *MC4R* is the most common single known cause of monogenic obesity [35]. *MC4R* was initially ident**i**fied as a gene predisposing to obesity and higher levels of BMI and fat mass [36]. Many studies which attempted to investigate the association between *MC4R* and type 2 diabetes risk gave inconsistent results, including those conducted in Chinese populations [10]–[14], [18], [23], [24], [26]. For example, two studies reported that rs17782313, which is in linkage disequilibrium with rs12970134, was associated with type 2 diabetes (OR = 1.20) [14], [29]. However, other studies in Chinese populations did not observe this association [12], [13], [30]. The discrepancy could partly be explained by limitations in sample size or sampling bias. In the present study, we identified that rs12970134 was associated with type 2 diabetes regardless of BMI and WC adjustment and had a similar effect size in Asians and Caucasians [13], [18], [26], [27], [36]–[38], suggesting a trans-ethnic replication of the effect of *MC4R* on type 2 diabetes. The further meta-analysis combining previous and our current findings supported the above finding and gave a more precise estimate of the effect of *MC4R* on type 2 diabetes in Chinese by raising the sample size to 15 462 diabetic patients and 13 865 controls. In addition, our results were in accordance with a previous meta-analysis involving multiethnic populations by Xi et al [10].Notably, a recent study in Japanese found that rs17782313 was related to glycated hemoglobin, which could be used in type 2 diabetes screening in populations independent of BMI, indicating the potential value of *MC4R* in type 2 diabetes prediction [39].

*GNPDA2* encoding glycosamine-6-phosphate deaminase 2 is highly expressed in the hypothalamus, but the biologic function of its gene product has not been clearly elucidated. The association between the G allele of rs10938397 and increased BMI has been well established by GWAS [28], while its relationship with type 2 diabetes is not clear [23]–[26]. Previous studies focused on the association between rs10938397 and type 2 diabetes in Chinese populations provided inconsistent observations [11]–[13]. In the present study, the association between the G allele of rs10938397 and the increased risk for type 2 diabetes attenuated after BMI and WC adjustment. Further meta-analysis involving previous and current studies in Chinese populations supported the findings of the current study, which suggests the effect of *GNPDA2* on type 2 diabetes risk may be mediated through obesity.

*BAT2*, also known as *PPRC2A*, is located within human major histocompatibility complex class III region with unclear gene function. The T allele of SNP rs2260000 within *BAT2* was identified as being associated with higher body weight and BMI in a previous GWAS [37] and the present study. An interesting finding of this study is that the obesity-related T allele is a protective allele for type 2 diabetes. A similar finding has been reported by a previous study in a Danish population with marginal significance [25]. These findings suggest that the effect of *BAT2* on type 2 diabetes may not be mediated by insulin resistance caused by obesity. The *BAT2* gene has been reported to be associated with insulin-dependent diabetes and thought to be involved in the inflammatory process of β-cell destruction [40]. Another potential mechanism could be that *BAT2* may play a role in the maintenance of glucose homeostasis via regulation of glucose-fatty acid conversion, which may lead to lower blood glucose and increased adiposity at the same time. Functional studies are warranted to reveal the biological function of the *BAT2* gene and SNP rs2260000 in the pathogenesis of obesity and type 2 diabetes.

The present study has several strengths. First, it is the largest study to investigate the associations between obesity-related GWAS loci and type 2 diabetes in the Chinese population. Second, all the study subjects are Han Chinese who are genetically homogeneous. Third, because central obesity could play a role in diabetes independent of BMI, we included adjustment of WC in addition to BMI in regression models. Finally, we performed meta-analysis that combined previous studies and present findings to provide more precise estimates for the effect of *MC4R* and *GNPDA2* on the risk for type 2 diabetes. However, our study still has limitations. We only tested one SNP from most of the genomic loci, which may lead to negative findings due to the lack of good coverage of the regions. We didn't correct for multiple testing. However, our study is hypothesis-driven and correction for multiple testing tends to be too conservative. In addition, the effects of obesity-related loci on quantitative glycemic traits in the control group without diabetes may not be good estimates for their effects in the general population. Especially, some of the risk alleles for dysregulated glycemic traits were not the reported risk allele for obesity. Further studies are warranted to replicate these findings and clarify the function implication of these loci.

In conclusion, the present study identified several obesity-related genomic loci associated with type 2 diabetes and glycemic-related traits in a large Chinese population. Among those, genetic loci near *MC4R* and within *BAT2* have effects on type 2 diabetes independent of obesity. The effect of the genetic locus including *GNPDA2* on type 2 diabetes might be mediated through obesity. The present study may help enhance the understanding of obesity in the pathogenesis of type 2 diabetes. In addition, it provides novel insights into the role of obesity-related genomic loci in the risk of type 2 diabetes beyond insulin resistance.

## Supporting Information

Table S1 Information of genotyped SNPs. Abbreviations: SNP, single nucleotide polymorphism; Chr, chromosome; T2D, type 2 diabetes; EU, European; HB, Han Chinese; BMI, body mass index; WHR, waist-hip ratio; WC, waist circumference. ^a.^ Risk allele for obesity is underlined. ^b.^ Genotype distributions are shown as the counts of three genotypes (bb, Bb, BB). B, major allele; b, minor allele.
(DOCX)

Table S2 The joint effects of obesity-related SNPs on glycemic quantitative traits in control subjects. Abbreviations: OGTT, oral glucose tolerance test; ISIm, Matsuda index; BMI, body mass index; WC, waist circumference. All non-Gaussian distributed quantitative traits in the control group were natural logarithmically transformed to normalize distributions. *β* value is reported for genotype risk score in multivariable regression models: model 1, adjusted for age and sex; model 2, adjusted for age, sex and BMI; model 3, adjusted for age, sex, BMI and WC.
(DOCX)
