# Supplementary material for: Obesity-Related Genomic Loci Are Associated with Type 2 Diabetes in a Han Chinese Population
Source: PLoS One. 2014 Aug 5;9(8):e104486. doi: 10.1371/journal.pone.0104486 (PMC4122466; doi:10.1371/journal.pone.0104486)
Supplement: Table S1 — Information of genotyped SNPs. Abbreviations: SNP, single nucleotide polymorphism; Chr, chromosome; T2D, type 2 diabetes; EU, European; HB, Han Chinese; BMI, body mass index; WHR, waist-hip ratio; WC, waist circumference. a. Risk allele for obesity is underlined. b. Genotype distributions are shown as the counts of three genotypes (bb, Bb, BB). B, major allele; b, minor allele. (DOCX) [file pone.0104486.s001.docx]

**Table S1.** Information of genotyped SNPs.

| **SNP** | **Gene** | **Chr** | **Position (build 36.3)** | **Gene region** | **Major/**  **minor allele^a^** | **Minor allele frequency** | | **Genotype** | ***P*_HWE_** | **Minor allele frequency in HapMap** | | **Related traits reported** | |
| --- | --- | --- | --- | --- | --- | --- | --- | --- | --- | --- | --- | --- | --- |
|  |  |  |  |  |  |  |  | **(bb/Bb/BB)^b^** |  |  |  |  |  |
|  |  |  |  |  |  | **T2D** | **Control** | **Control** |  | **CEU** | **CHB** |  |  |
| rs2568958 | *NEGR1* | 1 | 72537704 | Intergenic | A/G | 0.082 | 0.085 | 34/709/3854 | 0.775 | 0.363 | 0.077 | BMI, obesity, weight | |
| rs10913469 | *SEC16B* | 1 | 176180142 | Intron | T/C | 0.247 | 0.241 | 213/1647/2436 | **0.002** | 0.254 | 0.232 | BMI, weight | |
| rs2605100 | *SLC30A10* | 1 | 217710847 | Intergenic | G/A | 0.215 | 0.223 | 214/1618/2761 | 0.250 | 0.314 | 0.182 | Adiposity | |
| rs7561317 | *TMEM18* | 2 | 634953 | Intergenic | G/A | 0.093 | 0.100 | 34/860/3733 | 0.042 | 0.146 | 0.062 | BMI, weight | |
| rs7647305 | *ETV5/DGKG* | 3 | 187316984 | Intergenic | C/T | 0.064 | 0.069 | 16/568/3768 | 0.343 | 0.201 | 0.074 | BMI, weight | |
| rs10938397 | *GNPDA2* | 4 | 44877284 | Intergenic | A/G | 0.320 | 0.300 | 362/1838/2076 | 0.117 | 0.451 | 0.250 | BMI, obesity | |
| rs2260000 | *BAT2* | 6 | 31701455 | Intron | C/T | 0.481 | 0.492 | 1086/2334/116 | 0.215 | 0.606 | 0.485 | Weight | |
| rs4712652 | *PRL* | 6 | 22186594 | Intron | A/G | 0.143 | 0.147 | 110/1120/3341 | 0.174 | 0.429 | 0.124 | Adiposity (WHR) | |
| rs987237 | *TFAP2B* | 6 | 50911009 | Intron | A/G | 0.168 | 0.174 | 124/1205/2859 | 0.872 | 0.164 | 0.168 | BMI, adiposity | |
| rs545854 | *MSRA* | 8 | 9897490 | Intergenic | G/C | 0.419 | 0.421 | 795/2250/1512 | 0.412 | 0.177 | 0.348 | WC | |
| rs4923461 | *BDNFOS* | 11 | 27613486 | Intron | A/G | 0.431 | 0.435 | 844/2280/1434 | 0.252 | 0.230 | 0.442 | BMI, weight | |
| rs925946 | *BDNF* | 11 | 27623778 | Intergenic | G/T | 0.052 | 0.053 | 9/469/4140 | 0.304 | 0.323 | 0.033 | BMI, weight | |
| rs10838738 | *MTCH2* | 11 | 47619625 | Intron | A/G | 0.327 | 0.317 | 464/1975/2136 | 0.811 | 0.363 | 0.307 | BMI | |
| rs7138803 | *FAIM2* | 12 | 48533735 | Intergenic | G/A | 0.292 | 0.287 | 362/1912/2322 | 0.264 | 0.345 | 0.288 | BMI, obesity, weight, WC |  |
| rs1424233 | *MAF* | 16 | 78240252 | Intergenic | A/G | 0.327 | 0.329 | 507/2003/2082 | 0.442 | 0.442 | 0.307 | Obesity | |
| rs12970134 | *MC4R* | 18 | 56035730 | Intergenic | G/A | 0.209 | 0.189 | 170/1405/3035 | 0.631 | 0.279 | 0.179 | BMI, weight, WC | |
| rs1805081 | *NPC1* | 18 | 19394430 | Coding | A/G | 0.224 | 0.226 | 238/1600/2750 | 0.800 | 0.473 | 0.234 | Obesity | |
| rs29941 | *KCTD15* | 19 | 39001372 | Intergenic | T/C | 0.241 | 0.243 | 239/1388/2211 | 0.292 | 0.680 | 0.274 | BMI, weight | |

Abbreviations: SNP, single nucleotide polymorphism; Chr, chromosome; T2D, type 2 diabetes; EU, European; HB, Han Chinese; BMI, body mass index; WHR, waist-hip ratio; WC, waist circumference.

^a.^ Risk allele for obesity is underlined.

^b.^ Genotype distributions are shown as the counts of three genotypes (bb, Bb, BB). B, major allele; b, minor allele.
