# Supplementary material for: Obesity-Related Genomic Loci Are Associated with Type 2 Diabetes in a Han Chinese Population
Source: PLoS One. 2014 Aug 5;9(8):e104486. doi: 10.1371/journal.pone.0104486 (PMC4122466; doi:10.1371/journal.pone.0104486)
Supplement: Table S2 — The joint effects of obesity-related SNPs on glycemic quantitative traits in control subjects. Abbreviations: OGTT, oral glucose tolerance test; ISIm, Matsuda index; BMI, body mass index; WC, waist circumference. All non-Gaussian distributed quantitative traits in the control group were natural logarithmically transformed to normalize distributions. β value is reported for genotype risk score in multivariable regression models: model 1, adjusted for age and sex; model 2, adjusted for age, sex and BMI; model 3, adjusted for age, sex, BMI and WC. (DOCX) [file pone.0104486.s002.docx]

**Table S2.** The joint effects of obesity-related SNPs on glycemic quantitative traits in control subjects.

| **Traits** | **Model 1** | | | **Model 2** | | | **Model 3** | | |
| --- | --- | --- | --- | --- | --- | --- | --- | --- | --- |
|  | ***β*** | **SE** | ***P*** | ***β*** | **SE** | ***P*** | ***β*** | **SE** | ***P*** |
| Fasting plasma glucose | 0.0003 | 0.0008 | 7.03E-01 | 0.0001 | 0.0008 | 8.56E-01 | 0.0002 | 0.0008 | 7.48E-01 |
| 30-min OGTT glucose | -0.0005 | 0.0017 | 7.68E-01 | -0.0005 | 0.0017 | 7.80E-01 | -0.0004 | 0.0017 | 8.17E-01 |
| 2-h OGTT glucose | -0.0012 | 0.0015 | 4.26E-01 | -0.0016 | 0.0015 | 2.95E-01 | -0.0013 | 0.0015 | 3.90E-01 |
| Fasting serum insulin | -0.0009 | 0.0036 | 8.03E-01 | -0.0024 | 0.0036 | 5.10E-01 | -0.0020 | 0.0036 | 5.72E-01 |
| 30-min OGTT insulin | 0.0019 | 0.0057 | 7.31E-01 | 0.0001 | 0.0056 | 9.80E-01 | -0.0001 | 0.0056 | 9.89E-01 |
| 2-h OGTT insulin | -0.0044 | 0.0055 | 4.26E-01 | -0.0067 | 0.0055 | 2.20E-01 | -0.0062 | 0.0054 | 2.57E-01 |
| HOMA-B | 0.0025 | 0.0047 | 5.95E-01 | 0.0013 | 0.0047 | 7.78E-01 | 0.0014 | 0.0047 | 7.72E-01 |
| Insulinogenic index | -0.0001 | 0.0088 | 9.90E-01 | -0.0030 | 0.0087 | 7.33E-01 | -0.0038 | 0.0087 | 6.61E-01 |
| HOMA-IR | -0.0010 | 0.0038 | 7.85E-01 | -0.0026 | 0.0037 | 4.91E-01 | -0.0022 | 0.0037 | 5.64E-01 |
| ISIm | 0.0026 | 0.0037 | 4.80E-01 | 0.0039 | 0.0036 | 2.81E-01 | 0.0036 | 0.0036 | 3.24E-01 |

Abbreviations: OGTT, oral glucose tolerance test; ISIm, Matsuda index; BMI, body mass index; WC, waist circumference.

All non-Gaussian distributed quantitative traits in the control group were natural logarithmically transformed to normalize distributions.

*β* value is reported for genotype risk score in multivariable regression models: model 1, adjusted for age and sex; model 2, adjusted for age, sex and BMI; model 3, adjusted for age, sex, BMI and WC.
